# A Manual of Medical Jurisprudence

**Published:** 1844-04-01

**Authors:** 


### A MANUAL OF MEDICAL JURISPRUDENCE. By *Alfred S. Taylor*, Lecturer on Medical Jurisprudence and Chemistry in Guys's Hospital. 8vo. pp. 693. London : John Churchill.

WE have seldom had occasion to exercise our critical functions with greater satisfaction to ourselves than in the instance of the work which we now introduce to the notice of our readers. The only objectionable thing connected with Mr. Taylor's book is its name, and even here the objection weighs in favour of the book itself, which is not, as the title-page would induce us to believe, a "manual," but the most elaborate and complete treatise on legal medicine that has hitherto issued from the press.

In a very well-written and appropriate preface, the author indicates the general bearings of his subject. We recommend the concluding remarks to the special attention of those practitioners who either depreciate the importance of the study of legal medicine, or indulge their indolence in neglecting its cultivation with the hope that they may never be called into a court of law as medical witnesses, or may escape grave responsibility should such an undesired event occur.

The various subjects treated of in the body of the work have been taken up in the order of their importance, and the proportionate space occupied by each has been allotted on the same principle. More than one third of the entire volume is devoted to practical toxicology, which of all departments of medical jurisprudence most frequently puts the knowledge of the medical practitioner into requisition.

In Chap. I. our author discusses the question "what is a poison?" He properly objects to the common definition of a poison as a substance which, when administered in small quantity, is capable of acting deleteriously on the body—a definition which would exclude many substances universally admitted to be poisons—especially some belonging to the irritant class. The following observations include Mr. Taylor's own definition.

"In legal medicine, it is difficult to give such a definition of a poison as shall be entirely free from objection. Perhaps the most comprehensive definition which can be suggested is this: 'A poison is a substance which, when taken internally, is capable of destroying life without acting mechanically on the system.' Under this definition, it might be objected that the whole class of medicines, and numerous substances of an inert nature, would be included. Thus it is well known, that there are many cases on record in which cold water, swallowed in large quantity, and in an excited state of the system, has led to the destruction of life either rapidly by shock, or slowly by inducing gastritis. Any cold liquid such as iced water, beer, or ice itself, may have an equally fatal effect. The action of water, or cold liquids, under these circumstances, cannot be said to be mechanical; it appears to be due to the shock suddenly induced on the nervous system through the lining membrane of the stomach, and yet it would be inconsistent to class these inert liquids among poisons. In all cases of this description, it appears to me, that we are justified in drawing the following distinction between poisonous and non-poisonous substances. If the deleterious effect does not depend upon the nature of the substance taken, but upon the state of the system at the time at which it was swallowed, the substance cannot be regarded as a poison. All poisonous substances are *per se* deleterious,—the state of the system, setting aside for the present the peculiar effects of idiosyncracy and habit, has very little influence on their operation. The symptoms may be suspended for a time or slightly modified in their progress, but, sooner or later, the poison will affect the healthy and diseased, the old and the young, with a uniformity in its effects, not to be easily mistaken. A distinction of this kind, cannot however, be drawn except by a professional man, who has given attention to the subject of toxicology, and therefore it is no matter of surprise that poisoning should have been in more than one instance erroneously imputed, in cases where death has followed the drinking of cold liquids. In thus giving the medical definition of a poison, it is necessary to observe that the law never regards the manner in which the substance administered acts. If it be capable of injuring the health of an individual, it is of little consequence, so far as the responsibility of a prisoner is concerned, whether its action on the body be of a mechanical or chemical nature. Thus a substance which simply acts mechanically on the stomach, may, if wilfully administered with intent to injure, involve a person in a criminal charge, as much as if he had administered arsenic, or any of the ordinary poisons. It is then necessary that we should consider what the law means by the act of poisoning. If the substance criminally administered destroy life, whatever may be its nature or mode of operation, the accused is tried on a charge of murder or manslaughter, and the whole duty of the medical witness consists in showing that the substance taken was the certain cause of death. If, however, death be not a consequence, then the accused is tried under a particular statute for the attempt to murder by poison (1 Vict. c. 85, sec. 2). The words of this statute are very general, and embrace all kinds of substances, whether or not they be popularly or professionally regarded as poisons. Thus it is laid down that: 'Whosoever shall administer or cause to be taken by any person, any poison or other destructive thing with intent to commit murder, shall be guilty of felony, and being convicted thereof shall suffer death.' The same administering with intent, &c., although no bodily injury be effected, is felony, punishable by transportation for life, for fifteen years, or imprisonment for any term not exceeding three years. Such is the present state of the law of England in respect to attempts at poisoning where death does not take place. While the words of the statute render it unnecessary for a medical witness, in such cases, to give judicially a very close definition of a poison; they impose upon him a difficulty which he must prepare himself to meet. The substance administered may not be a poison in the medical signification of the term, nor may it be popularly considered as such, and yet, when taken, it may be destructive to life. We have examples of substances of this description in iron filings, powdered glass, pins and needles, and such like bodies, all of which have been administered with the wilful design of injuring, and have, on various occasions, given rise to criminal charges. In cases of this kind, the legal guilt of a prisoner may often depend on the meaning assigned by a medical witness to the words *destructive thing*. Thus, to take an example,—liquid mercury might be poured down the throat of a young infant, with the deliberate intent to destroy it. A question of a purely medical nature will then arise, whether mercury be a destructive thing or not; and the conviction of the prisoner will probably depend on the answer returned by the witnesses. Should a difference of opinion exist, an occurrence by no means unusual in medical evidence, the prisoner will, according to the humane principle of our law, receive the benefit of the doubt." 5—7.

The foregoing extract may serve as an example of the plain, sensible, and practical manner in which our author treats his subjects. We are not quite content with his *definition* of a poison, because it will not apply to those poisons which are innocuous when taken into the stomach, but deadly when inserted beneath the skin, as the venom of serpents. It appears to us that a definition of a poison, to be accurate, must consist of two members. It would not be enough to say "a poison is a substance which, being taken into the stomach, or otherwise introduced into the living system, occasions death independently of any mechanical injury," because it might hence be inferred that it was indifferent, with respect to the poisonous operation of any substance, whether it were taken into the stomach, or introduced into the system, in any other way—which is not the fact. If we were asked to define a poison, we should do it thus:—"Every substance which, being taken into the stomach, is capable of causing death independently of mechanical injury, is a poison; and every substance which, being introduced into the living system, though otherwise than by the stomach, if capable of causing death independently of mechanical injury, is also a poison."

In the second chapter, on "the mode of action of poisons," our author has followed Orfila and Christison, adding, however, what was needful to bring his information quite up to the present time. Chap. III. is on "the classification of poisons," and that adopted is a modification of Orfila's, with what appears to us an improvement in the subdivision of the class of irritants. The contents of Chap. IV. are extremely valuable, and in a great measure new. They relate to "the rules to be observed in investigating a case of poisoning," and constitute an excellent guide to the practitioner in inquiries which are frequently among the most difficult in which he can be engaged, and which require much forethought, complete knowledge of the subject, and no small sagacity and readiness in applying that knowledge. Chapters V. to IX. inclusive, treat of "the evidence of poisoning in the living subject"—"the evidence of poisoning in the dead subject"—"the evidence of poisoning from chemical analysis"—"the evidence of poisoning from experiments on animals"—and the question, "was death caused by poison?"

Adopting the general plan laid down by Orfila, and followed by Christison, Mr. Taylor has illustrated these important subjects with a great variety of cases, many of which have never appeared in any preceding work on legal medicine, while his own observations on these cases, which are of the most pithy and practical character, give them a direct and useful bearing on the method of actually conducting medico-legal inquiries relating to poisoning. Chap. X. contains some "concluding remarks on general poisoning," and the following valuable statistical statements of the relative frequency of death from different poisons in England, Denmark, and France.

"In concluding this chapter," says Mr. Taylor, "I wish to call the attention of the reader to some facts connected with the statistics of poisoning. In relation to medico-legal practice, this is a subject of some interest; because it will indicate to the medical jurist what are the poisons that are most frequently selected for the purposes of suicide and murder, and with the properties of which it will be expected that he should be acquainted. Unfortunately very few tables of this kind have been published; and those which have appeared are defective in many points. One of the best is that which was published some years since from the returns made by the coroners of England, of the number of inquisitions held in the years 1837 and 1838, wherein death was caused by poison. The following is an abstract of the paper, which appeared in the Medical Gazette for November, 1839.

"The number of deaths by poison (returned) in the two years above-mentioned were 541, of which number 282 were males, and 259 females. The substances which caused death, may be taken in the following numerical order.

| | | | |
|---|---|---|---|
| Opium. | | | |
| Laudanum | 133 | | |
| Opium | 42 | | |
| Other preparations | 21 — | 196 | |
| Arsenic | | 185 | |
| Sulphuric acid | | 32 | |
| Prussic acid | | 27 | |
| Oxalic acid | | 19 | |
| Corrosive sublimate and mercury | | 15 | |
| Mixed or compound poisoning | | 14 | |
| Oil of bitter almonds | | 4 | |
| Poisonous mushrooms | | 4 | |
| Colchicum, nux vomica (of each 3) | | 6 | |
| Nitric acid, caustic alkali, tartar-emetic, acet. morphia, strychnia, deadly nightshade, aconite, (of each 2) | | 14 | |
| Bichrom. potash, nit. silver, Goulard's extract, sulph. iron, mur. tin, hellebore, castor-oil seeds, savin, hemlock, cantharides, cayenne pepper, (of each 1) | | 11 | |
| | | | 527 |
| Unknown | | | 14 |
| | | | 541 |

"It will be seen by this table, that the largest proportion of cases of poisoning in England are those by opium and arsenic: the greater number of the former being cases of suicide and accident, and of the latter, cases of criminal poisoning. The two next are tables, the one of the statistics of poisoning in Denmark, from the year 1830 to 1835, (Medical Gazette, January, 1840); the other of about an equal number of cases observed in France, (Journal de Chemie Med. 1835.)

*In Denmark.*

| | |
|---|---|
| Sulphuric or nitric acid generally diluted | 74 |
| Arsenic | 16 |
| Caustic alkalies | 5 |
| Opium | 2 |
| Litharge, verdigris (of each 1) | 2 |
| | 99 |

*In France.*

| | |
|---|---|
| Arsenic | 58 |
| Verdigris | 7 |
| Corrosive sublimate | 6 |
| Cantharides | 5 |
| Nux vomica | 4 |
| Nitric acid | 2 |
| Sulphuric acid, acet. lead, carb. lead, sulph. zinc, tartar emetic, opium, (of each 1) | 6 |
| | 88 |

"It is difficult to compare these two tables with the preceding—the number of observations being much fewer. Poisoning by arsenic is, however, proved to be very common. It is remarkable that the mineral acids should have caused so many deaths in the kingdom of Denmark, the proportion being no less than three-fourths of the whole number." 83—4.

The succeeding eighteen chapters are occupied with the individual poisons, the symptoms they excite, the treatment best calculated to obviate their effects, the means of detecting their presence, and other topics.

From the many discoveries and improvements recently made in toxicology, a revisal of the old processes for the detection of poisons was required, and our author, in conducting this task, has succeeded admirably in simplifying them, and giving certainty to their results.

Chap. XXII. and Appendix A. afford sufficient evidence of the success of his labours. They are manifestly the work of a highly-informed and thoroughly practical chemist and toxicologist, and by attending to the rules in Chap. XXII., the nature of a large number of poisons may be distinguished by a few simple tests, and in a few minutes' time. A material point for a medical witness is, that he should know what *objections* may be urged against the evidence founded on the chemical analysis of poisonous liquids. This point has hitherto been little attended to in works on legal medicine; Mr. Taylor, however, has, with great judgment, devoted a section, under the head of each poison, to the special consideration of all such objections, and pointed out the proper answer to each. The mode of determining the quantity of poison present in a suspected liquid—a question sometimes of great importance with reference to the presence or absence of malice in the act of poisoning—is also particularly explained; and attention has been given to the smallest quantity of each poison that has been known to destroy life, as well as the largest quantity from the effects of which a person has recovered. The longest and the shortest period within which each poison has been known to destroy life, has likewise been carefully adverted to.

Chapters XXIX. to XLI. inclusive, are devoted to the extensive subject of wounds, which is throughout treated in the most masterly manner. As an example, we would willingly transfer to our pages the entire chapter on gun-shot wounds, but our space will not admit of this, and any attempt to condense Mr. Taylor's observations would be doing them injustice: we will therefore content ourselves with extracting the following passage relating to the important and sometimes difficult inquiry as to the distance at which the piece which has inflicted a wound has been fired.

"The question whether a piece was fired near to, or at a distance from the wounded party, may become of material importance on a charge of homicide. Two persons may quarrel, one having a loaded weapon in his hand, which he may allege to have been accidentally discharged and to have killed the deceased. If the allegation be true, we ought to find on the body the marks of a near wound: if, however, it were such as that it had been produced from a distance, and therefore after the quarrel,—the medical proofs of this fact might imply malice, and involve the accused in a charge of murder. The following case occurred in Ireland in 1834:—A tithe-collector was tried for the murder of a man, by shooting him. It appeared in evidence, that the prisoner, while on duty, was attacked by the deceased and two of his sons, and he drew a pistol to intimidate them. He was dragged off his horse by these parties, and during the scuffle, it is supposed, the pistol accidentally went off, and inflicted a wound on the deceased, of which he died shortly afterwards. The sons of the deceased swore that the prisoner, when at some distance, took a deliberate aim, and fired the pistol at their father; and a priest came forward to depose, that such was the dying declaration of the deceased. From some subsequent suspicion of the truth of this story, the body, which had not been properly inspected in the first instance, was ordered to be disinterred. It was carefully examined by a surgeon, who was enabled to swear positively, that the pistol must have been fired close to the body of the deceased, and not at a distance; since there were the marks of powder and burning on the wrist. Hence it clearly followed that the pistol had been discharged during the scuffle, either by accident or in self-defence. The prisoner was acquitted, and the parties who had appeared as witnesses against him, were indicted and convicted of perjury. In the case of Mr. Pearce, a surgeon, who was tried at the Central Criminal Court, in 1840, for shooting his wife, and was found insane, it appeared from the medical evidence that the pistol had been fired so near the person of the prosecutrix, that her dress was burnt and the skin blistered. Mr. Marshall relates that, when stationed at Ceylon with troops, a man who had but recently joined the regiment, was placed as sentry in a position, where he was occasionally fired at by the enemy from the surrounding jungle. The man was one day found severely wounded; the calf of his leg was greatly torn, the whole charge of a musket having passed through it. He attributed the wound to a shot from the enemy, but, from the skin of the leg being completely blackened by charcoal, it was clear that it must have arisen from the discharge of his own musket. He had inflicted this wound upon himself, in order to obtain a discharge from the regiment. These examples then show, that both the dress and skin of a person who has received a gun-shot wound should be closely examined. The result may be, that the statement given of the mode in which it was received, will be entirely disproved. The case of M. Peytel, tried in France, in September 1839, presents many points of great interest in relation to the medical jurisprudence of gun-shot wounds. This gentleman was travelling in a carriage, in company with his wife and attended by a man-servant. The wife and the man-servant were found dead, on the road, and the account given by M. Peytel, was, that the servant had discharged a pistol into the carriage, and shot his wife, and he had afterwards pursued and killed him. The facts, however, were so suspicious against M. Peytel, that he was charged with the double murder. From an examination of the body of the wife, it appeared that there were two pistol wounds in the face, which had most probably been produced by two separate pistols. The prisoner alleged, that about nine o'clock at night, when it was dark, he desired the servant to get down in order to relieve the horses. Two minutes afterwards, some man, whom he afterwards found to be the servant, approached the carriage-door, discharged a pistol at him, and wounded his wife; but the evidence showed that two weapons must have been used, or at least two different discharges made by a person sitting very near to the deceased, so that the muzzles must have almost touched her face, the eye-lashes and skin having been much burnt by the powder. These facts, together with other strong circumstances against him, led to the prisoner's conviction. Dr. Ollivier, who appeared in the prisoner's favour, considered that the deceased might have been shot by the servant, and that the two wounds might have been produced by one pistol loaded with two bullets; also, that the marks of burning about the face of the deceased, might be attributed to the wadding, and therefore they afforded no proof that the muzzle of the pistol had, at the time of its discharge, been close to her person. He also contended that the deceased had not died from the wounds. Notwithstanding these ingenious medico-legal arguments, there can be no doubt that the prisoner was very properly convicted (See Ann. d'Hyg. 1839, p. 339). It has been said, that when a bullet is fired near, it commonly traverses; and therefore it has been rather hastily assumed, that where there is only one external wound, and the bullet has lodged, there is a proof that the piece has been fired from a distance. This inference is, however, erroneous. A bullet may be fired close to the person, and yet not traverse the body, either from its impulsive force not being sufficiently great, or from its meeting with resistance in the body. Many cases might be cited to show, that in the near wounds produced by suicides and murderers, the bullets have not always traversed the body. In suicide, when the piece is discharged into the mouth, the projectile often lodges in some part of the cranium. In the late assassination of Mr. Drummond, the pistol was discharged close to the back of the deceased; the ball, however, had not traversed, it had lodged beneath the skin in the fore-part of the abdomen. It is then, it appears to me, out of the power of a witness to say, from the mere fact of a bullet lodging or traversing, whether the assassin was far off or near at the time the deceased was wounded. The latter point may sometimes be readily determined by the marks of injury and burning to the skin and dress." 411-12.

After a good chapter on burns and scalds, we come to the subject of infanticide, which occupies eight chapters. All the medico-legal questions connected with this subject are separately examined, and especial attention is given to the proofs of life and the causes of death—the two most important points in a case of child-murder. Chapters XLV. XLVI. and L. we may refer to in particular, as being full of valuable matter. In the first of these chapters the hydrostatic test is very fully commented upon, and the many erroneous opinions with regard to it are confuted by a reference to conclusive facts. The cases in which this test will really assist a medical jurist, and those in which it fails to do so, are indicated as clearly as the nature of the subject will admit. At the conclusion of this, and of the succeeding chapter, the author shows, in a striking point of view, the extraordinary position in which medical witnesses are placed by the legal doctrines now prevalent on the subject of infanticide.

Chapter LI. is on "Drowning." We would here particularly direct the attention of the reader to the observations relative to the marks of violence on the bodies of drowned persons, which contain some matter that is both new and practical. In Chap. LII. on "Hanging," the results of Casper's experiments will be found, with some valuable observations on the mark produced by the cord. The subject of "strangulation" is usually considered in connexion with that of hanging, but has here a separate chapter (LIII.) devoted to it. Chap. XLIV. on suffocation by carbonic acid, contains many original experiments and observations, and, together with the succeeding one, on the effects of sulphuretted hydrogen, drains, sewers, coal gas, and mechanical smothering, forms an admirable exposition of all those medico-legal questions in which asphyxia is involved.

The subjects of the next chapter, "lightning, cold, starvation," afford little scope for original remark.

Chapters LVII. and LVIII. on rape, pregnancy, and delivery, contain a full illustration of every point connected with these subjects. In Chap. LIX. on concealment of birth, and criminal abortion, some new facts and observations will be found relative to what constitutes abortion in law. The remarks in p. 590 are of great practical value. The two succeeding chapters treat of birth, inheritance, gestation, premature births, protracted births, and paternity, and present some new and interesting medico-legal information. In Chap. LXII. the subjects of superfœtation, impotence, and sterility, are briefly, but sufficiently commented upon. The remaining four chapters are devoted to "insanity," and comprehend everything of importance relative to that state. The medical and legal tests of insanity, civil and criminal responsibility, restraint, interdiction, lucid intervals, and every point connected with the subject of unsound mind, which claims the attention of the medical jurist, are viewed in a strictly practical light, and with a direct reference to the conduct of medical witnesses in courts of law.

In an Appendix, we find a list of tests and apparatus required for the analysis of poisons, to which we have already referred, and an abstract of the Medical Witnesses Act.

In conclusion, we recommend Mr. Taylor's work to all our readers as the ablest, most comprehensive, and, above all, the most practically useful book which exists on the subject of legal medicine. Even Beck's, which has so long and deservedly held chief possession of the field, must now give way to a work which is more copious and fresher in its information, though smaller in its dimensions, and lower in its price. Any man of sound judgment who has mastered the contents of Taylor's Medical Jurisprudence, may go into a court of law with the most perfect confidence of being able to acquit himself creditably.

